# Detecting epistatic interactions contributing to human gene expression using the CEPH family data

**DOI:** 10.1186/1753-6561-1-s1-s67

**Published:** 2007-12-18

**Authors:** Hua Li, Guimin Gao, Jian Li, Grier P Page, Kui Zhang

**Affiliations:** 1 Bioinformatics Center, Stowers Institute for Medical Research, 1000 East 50th Street, Kansas City, Missouri 64110, USA; 2 Section on Statistical Genetics, Department of Biostatistics, RPHB 327, 1530 3rd Avenue South, University of Alabama at Birmingham, Birmingham, Alabama 35294, USA; 3 Virginia Bioinformatics Institute, Virginia Tech, Blacksburg, VA 24061, USA

## Abstract

It is believed that epistatic interactions among loci contribute to variations in quantitative traits. Several methods are available to detect epistasis using population-based data. However, methods to characterize epistasis for quantitative traits in family-based association analysis are not well developed, especially for studying thousands of gene expression traits. Here, we proposed a linear mixed-model approach to detect epistasis for quantitative traits using family data. The proposed method was implemented in a widely used software program SOLAR. We evaluated the power of the method by simulation studies and applied this method to the analysis of the Centre d'Etude du Polymorphisme Humain family gene expression data provided by Genetics Analysis Workshop 15 (GAW15).

## Background

With the ability to measure simultaneously thousands of gene expression traits, understanding the causes of transcriptional variation has been of great interest. Genetic interactions, also called epistasis, have been shown to affect gene expression phenotypes. For example, Brem and Kruglyak [1] found that the genetic basis of transcripts in yeast is more often likely to be polygenic rather than monogenic and that in yeast epistasis effects are present in more than 15% of transcripts. Therefore, it is essential to analyze epistatic interactions between loci that contribute to variations in gene expression traits.

Several statistical methods for studying epistatic interactions between loci for quantitative traits using populations of unrelated individuals or from experimental designs have been developed [2-6]. For quantitative traits using family-based samples (related individuals), epistatic testing has been incorporated into the variance-component linkage analysis and implemented in the software SOLAR [7]. However, epistatic detection on the basis of the linkage analysis can only locate the two interacting loci in wide confidence intervals and will have small power for data sets with small sample sizes, such as in the GAW15 (Genetic Analysis Workshop 15) CEPH (Centre d'Etude du Polymorphisme Humain) data set, which only contains 194 individuals. In this situation, association-based methods are expected to have higher power for detecting epistasis. A variety of approaches [8,9] that focus on association testing can be used to detect epistasis [3]. However, these are transmission-disequilibrium test (TDT)-based methods, which also have lower power in epistasis studies. Also, it is complicated to identify the best statistical model for the joint effects of loci including their interactions through model selection, particularly for analyzing thousands of gene expression traits for thousands of markers.

In this paper, we have extended the association-based linear regression model [2,3] by adding a random polygenic effect into the model to allow for familial data for epistasis detection of quantitative traits. The proposed linear mixed model was implemented in the widely used software program SOLAR [7], which calculates significance levels for each covariate, and performs covariate screening in the model. We applied the proposed method to a subset of the gene expression profiles in the CEPH data set as provided by GAW15.

## Methods

### Statistical methods

Based on the linear regression model of Cokerham and Zeng [2] (also see Cordell [3]), we propose a linear mixed model for detecting epistatic interactions for quantitative traits using family-based data:

y = *μ *+ *a*_1_*x*_1 _+ *d*_1_*z*_1 _+ *a*_2_*x*_2 _+ *d*_2_*z*_2 _+ *i*_*aa*_*x*_1_*x*_2 _+ *i*_*ad*_*x*_1_*z*_2 _+ *i*_*da*_*z*_1_*x*_2 _+ *i*_*dd*_*z*_1_*z*_2 _+ *Wβ *+ *v *+ *ε*.

This model assumes diallelic marker loci and that *y *is a normally distributed quantitative gene expression phenotype from related individuals, *W *is a vector of fixed covariates such as sex effects, *β *is the corresponding vector of coefficients, *v *is the random polygenic effect within a family, the vector of polygenic effects in each family follows multi-normal distribution N(0, 2A$\sigma_{f}^{2}$) where A is the kinship matrix and $\sigma_{f}^{2}$ is the variance associated with vectors of polygenic effects, *a*_*i *_and *d*_*i *_are the additive and dominant effects, and *x*_*i *_and *z*_*i *_are dummy variables related to the genotypes at the locus *i*. For example, for a diallelic locus, we might set *x*_*i *_= 1 and *z*_*i *_= -0.5 for genotype *BB*, *x*_*i *_= 0 and *z*_*i *_= 0.5 for genotype *Bb*, and *x*_*i *_= -1 and *z*_*i *_= -0.5 for genotype *bb*, respectively. *i*_*aa*_, *i*_*ad*_, *i*_*da*_, and *i*_*dd *_are additive-additive, additive-dominant, and dominant-dominant interaction effects between the two loci, respectively, corresponding to epistatic interaction effects, and *ε *is the residual error, following normal distribution N(0, $\sigma_{e}^{2}$). Significant interaction effects imply presence of epistasis.

To detect epistasis, for each gene expression phenotype, we ran Model (1) in SOLAR for each pair of single-nucleotide polymorphisms (SNPs) in the selected candidate regions (see Description of the data set for more details). The number of tests for each gene expression phenotype ranges from 6 to 820, depending on the marker density and size of the candidate regions selected for the epistasis search. For each gene expression phenotype, individual *p*-values were adjusted using false-discovery rate (FDR) under the general dependency assumption [10] within each phenotype. FDR-adjusted *p*-values equal to or less than 0.05 (FDR ≤ 0.05) are considered to be significant.

### Simulation study

We simulated a data set based on the pedigree structure from CEPH family data, which has 14 three-generation families of 194 individuals. We considered two unlinked diallelic markers in our analysis with allele frequency of 0.5. Marker genotypes for the grandparents were generated assuming Hardy-Weinberg equilibrium at each locus. Genotypes for parents and children were simulated conditional on their parental genotypes following Mendel's law. As an example we evaluated the power (true negative rate) and type I error (false-positive rate) of the proposed method in identifying additive-additive epistatic effect *i*_*aa*_. Phenotypes of each individual was generated based on the Model (1), where *a*_1 _= 0.2, *d*_1 _= 0.01, *a*_2 _= 0.2, *d*_2 _= 0.01, *i*_*ad *_= *i*_*da *_= *i*_*dd *_= 0, *β *= 0.1 (the vector W only contains sex), $\sigma_{f}^{2}=\sigma_{e}^{2}=0.5$. When evaluating power and type I error we set *i*_*aa *_= 0.7 and *i*_*aa *_= 0, respectively. These values were chosen based on the estimated values from analysis of selected 27 traits in the CEPH family data. We plotted receiver operating characteristic (ROC) curve by calculating specificity and sensitivity as we varied the nominal threshold for determining the significant epistasis, where:

1 - specificity = (false positive)/(true negative + false positive)

sensitivity = (true positive)/(true positive + false negative).

### Description of the dataset

The CEPH family data provided by GAW15 includes 3554 Affymetrix^® ^gene expressions measured for 194 individuals from 14 three-generation CEPH families. In addition, 2882 autosomal and X-linked SNPs were typed for these individuals.

The software package pedStat as distributed in Merlin version 1.0.1 [11] was used to check for Mendelian inconsistence, genotyping proportions, and heterozygosity of SNPs. SNP markers with minor allele frequencies less than 1% (equivalent to heterozygousity < 1.98%), markers with greater than 30% missing genotypes, and markers with only two of the three possible genotypes were removed from analysis, which left 2436 SNPs for our analysis.

We limited our analysis to the 27 gene expression phenotypes with the strongest linkage evidence of *cis *effects (Table 1 from Cheung et al [12]). Their Table 1 listed one to two peak markers for each phenotype, where a peak marker is the SNP with the most significant finding in the genome-wide association analysis (GWA) for this phenotype. Fourteen of the 27 gene expression phenotypes exhibited *cis *regulation (with *cis *peak markers) by the GWA analysis. For the phenotype *PPAT *there are two peak markers that point to both *cis *and *trans *regulation for this gene. For the remaining 12 phenotypes, the peak markers are *trans *markers. In our study, for each of the 27 phenotypes we selected a 15-Mb candidate region centered on the target gene location. If a *trans *peak marker was identified in the GWA analysis, we also selected an additional 15-Mb candidate region centred on that marker. We analyzed the epistatic effects for all possible combinations of the SNPs within the candidate regions.

## Results

### Simulated data

**Figure 1:**
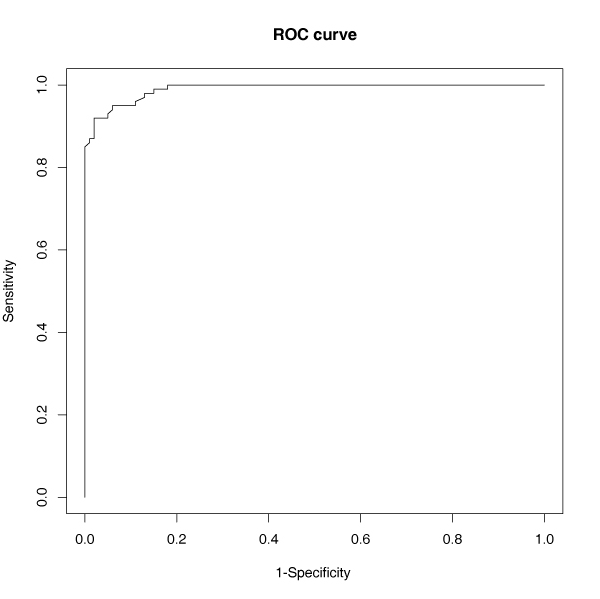
Receiver operating characteristic (ROC) curves for 100 simulated data.

We calculated the ROC profiles for detecting interacting SNPs using linear mixed Model (1). An ROC curve measures the trade-off between sensitivity (true-positive rate, TPR) and 1 - specificity (false-positive rate, FPR) for different cut-offs (Figure 1). Each threshold value results in a sensitivity and 1 - specificity, which is represented by a point on the ROC curve. For 100 simulated data sets, the TPR starts at 0.82 and quickly increases to 0.95, with FPR less than 0.11. When the nominal threshold is 0.05 for the significant calls of epistasis, the power is 0.95 and the type I error is 0.08 in identifying the additive by additive epistasis effect *i*_*aa*_. In summary, simulation results indicate the proposed linear mixed model has high sensitivity (power) and specificity for detecting SNP-pair interaction in family data for two unlinked markers.

### CEPH family data

When applying the linear mixed model to the analysis of the CEPH data, a total of six SNP pairs showed significant epistatic interactions for three gene expression phenotypes (Table 1). Two out of six significant epistatic effects are from SNPs that are located on two regions from different chromosomes. These two regions are corresponding to the *cis *linkage region and the *trans *association marker. These results show gene expression regulation cannot be simplified as *cis *or *trans *regulation because both *cis *or *trans *effects simultaneously contribute to variation in message RNA expression level. The strong epistatic interactions between SNPs could be interpreted as the strong epistatic interactions between genes if the nearby genes are in linkage disequilibrium (LD) with SNPs. For example, the SNP rs1537638 is within the gene *PTK7 *(protein tyrosine kinase 7), which is located on chromosome 6 and shows significant interaction with the SNP rs1505694, which is close to the gene *ITGB1BP1 *(integrin *β*1 binding protein 1) on chromosome 2. This suggests that these two genes might physically or genetically interact with each other or be involved in the same biological process. In fact, proteins encoded by these two genes are indeed involved in the cell adhesion process, in which *PTK7 *is a receptor tyrosine kinase transducing extracellular signals across the cell membrane and *ITGB1BP1 *plays an important role during integrin-dependent cell adhesion by binding the *β*1 integrin cytoplasmic domain.

**Table 1:** FDR adjusted *p*-values of significant epistatic effects detected under the full mode with the model selection between two loci located on different chromosomes in the analysis of the CEPH family data

Gene (location)	SNP1 (location)	SNP2 (location)	a_1_a_2_	a_1_d_2_	d_1_a_2_	d_1_d_2_
ITGB1BP1 (2p25.1)	rs1505694 (2p25.2)	rs1537638 (6p21.1)	**0.0291^a^**	**0.0092**	1	0.3725
TM7SF3 (12p11.23)	rs575030 11q23.3	rs725291 11q24.2	1	1	**0.0341**	**0.0387**
	rs725291 11q24.2	rs1944819 11q24.2	**0.0351**	1	**0.0341**	1
	rs725291 11q24.2	rs674237 11q24.2	0.0800	1	**0.0341**	1
	rs753013 12p11.22	rs1492332 12q12	1	1	**0.0341**	0.4308
PPAT (4q12)	rs1824965 (4q12)	rs39068 (7p15.1)	1	1	0.1377	**0.0351**

^a^Significant interaction effects are in bold.

## Conclusion and discussion

We have presented an association-based method for detecting epistatic interactions for quantitative traits using family data, and applied this method to the analysis of gene expression phenotypes of CEPH family data provided by GAW15. When we applied the proposed method to the CEPH data, we detected six SNP pairs that showed significant epistatic interactions for 3 gene expression phenotypes among the 27 phenotypes analyzed. We study the epistasis among genes by analyzing the interactions of SNPs located in the corresponding genes. This kind of epistasis detected from statistical tests does not necessarily correspond to the classic model of epistasis. Strong epistatic interactions among SNPs may not always indicate biological interactions among genes.

Although we demonstrated the association-based linear mixed-model approach for analyzing 27 phenotypes, the method is mainly proposed for analyzing thousands of phenotypes in genome-wide study. In general, one could identify two interacting linkage regions (QTL intervals) using two-dimensional genome linkage scan by allowing a higher a false-positive rate. Or one could do stepwise search of two interacting QTLs by identifying one primary QTL and then searching for the secondary QTL conditional on the primary locus being linked [13]. Once the two candidate intervals (regions) have been identified, the proposed linear mixed-model approach in this work could be used for epistasis detection between SNPS.

The proposed linear mixed model could be implemented in two steps by first regressing out the fixed effects (not including genetic effects) and polygenic effects and then detecting genetic interaction effects using predicted residuals from the first-step analysis. This strategy is attractive because of its flexibility in identifying the best statistical model for the joint effects of loci and computational efficiency for analyzing thousands of gene expression traits in genome-wide study.

## Competing interests

The author(s) declare that they have no competing interests.
